# HFSA-Net: A 3D Object Detection Network with Structural Encoding and Attention Enhancement for LiDAR Point Clouds

**DOI:** 10.3390/s26010338

**Published:** 2026-01-05

**Authors:** Xuehao Yin, Zhen Xiao, Jinju Shao, Zhimin Qiu, Lei Wang

**Affiliations:** 1 School of Transportation and Vehicle Engineering, Shandong University of Technology, Zibo 255000, China; 2 Shuntai Automobile Co., Ltd., Zibo 255400, China

**Keywords:** LiDAR, 3D object detection, deep learning, attention mechanism, multi-scale feature fusion

## Abstract

The inherent sparsity of LiDAR point cloud data presents a fundamental challenge for 3D object detection. During the feature encoding stage, especially in voxelization, existing methods find it difficult to effectively retain the critical geometric structural information contained in these sparse point clouds, resulting in decreased detection performance. To address this problem, this paper proposes an enhanced 3D object detection framework. It first designs a Structured Voxel Feature Encoder that significantly enhances the initial feature representation through intra-voxel feature refinement and multi-scale neighborhood context aggregation. Second, it constructs a Hybrid-Domain Attention-Guided Sparse Backbone, which introduces a decoupled hybrid attention mechanism and a hierarchical integration strategy to realize dynamic weighting and focusing on key semantic and geometric features. Finally, a Scale-Aggregation Head is proposed to improve the model’s perception and localization capabilities for different-sized objects via multi-level feature pyramid fusion and cross-layer information interaction. Experimental results on the KITTI dataset show that the proposed algorithm increases the mean Average Precision (mAP) by 3.34% compared to the baseline model. Moreover, experiments on a vehicle platform with a lower-resolution LiDAR verify the effectiveness of the proposed method in improving 3D detection accuracy and its generalization ability.

## 1. Introduction

As a core technology in the environmental perception systems of autonomous vehicles and intelligent robots, 3D object detection aims to determine the precise 3D spatial position, dimensions, and category of objects within a scene. This provides a critical basis for subsequent decision-making, planning, and control [1,2]. Among the various 3D sensors available, Light Detection and Ranging (LiDAR) has become the predominant modality for developing advanced 3D object detection algorithms, owing to its capacity to directly acquire precise 3D geometric information and its excellent robustness to changes in illumination [3].

Despite the significant progress achieved in LiDAR-based detection methods [4,5,6], their performance remains constrained by the intrinsic characteristics of point cloud data [7,8]. By nature, a LiDAR point cloud is a sparse and unordered sampling of scene surfaces, which gives rise to two fundamental challenges [9,10]. The first is data sparsity: due to the discrete scanning mechanism of LiDAR sensors, the collected data is inherently non-continuous and unevenly distributed, especially for distant or small objects, which are composed of only a minimal number of points, this sparsity leads to an inadequate feature representation. The second is the fragility of geometric structural information. To enhance computational efficiency, conventional methods widely adopt voxelization to convert the point cloud into a regular grid representation. However, this process is often accompanied by the degradation of fine-grained local geometric structures, causing an irreversible loss of information.

The information loss issue, stemming from the physical characteristics of sensors and data preprocessing pipelines, constitutes a significant performance bottleneck in current LiDAR-based 3D object detection, particularly for distant and small objects. The degradation of geometric details directly leads to a reduction in detection accuracy and an increase in the miss detection rate, posing a potential threat to the safety of autonomous driving systems. Therefore, developing an algorithmic framework that can effectively preserve and enhance the structural information of point clouds is of great scientific significance and practical value for advancing the field.

To address the aforementioned challenges, this paper proposes an enhanced 3D object detection network, namely HFSA-Net (Hierarchical Focus and Structural-Aware Network). This framework, an advancement based on the CenterPoint architecture, systematically resolves the information loss problem in sparse point clouds in a synergistic manner by incorporating three specially designed modules. The main contributions of this paper are summarized as follows:

(1) A structured voxel feature encoder is proposed, which explicitly compensates for the loss of local geometric information during voxelization by employing intra-voxel feature refinement and multi-scale neighborhood context aggregation. This is designed to improve the model’s representation capability for fine-grained structures.
(2) A hybrid-domain attention-guided sparse backbone network is constructed. This network introduces a decoupled hybrid-domain attention mechanism that enables the network to dynamically focus on salient feature regions within sparse point clouds, thereby enhancing the effectiveness of feature extraction.
(3) A scale-aggregated detection head is designed to enhance the model’s perception and localization capabilities for objects at varying distances and sizes. It achieves this by fusing a multi-level feature pyramid to adapt to variations in point cloud density.

Comprehensive experimental evaluations of the proposed model were conducted on the public KITTI dataset. The results demonstrate that the proposed algorithm achieves significant performance improvements across multiple metrics. Furthermore, extensive ablation studies and on-vehicle tests have verified the effectiveness of each innovative module and the practical utility of the framework.

## 2. Related Work

Existing 3D object detection algorithms can be primarily categorized into three main streams based on their distinct approaches to processing LiDAR point cloud data: point-based, voxel-based, and point-voxel fusion methods [11,12]. This section will review these mainstream technical routes, analyzing their respective advantages and limitations in handling point cloud information.

### 2.1. Point-Based Methods

Point-based methods operate directly on the raw point cloud set, aiming to fully preserve the geometric information of the scene. PointNet [13] and its successor, PointNet++ [14], pioneered this direction by designing symmetric network architectures capable of directly processing unordered point sets. These methods enable end-to-end learning on point clouds and can capture hierarchical local features. Building upon this foundation, PointRCNN [15] learns per-point features through set abstraction and other operations to generate high-quality 3D proposals.

In principle, this category of methods can retain the original information to the maximum extent, thus often achieving higher accuracy on tasks that require fine-grained geometric features. However, due to the necessity of processing large-scale point sets and performing complex neighborhood query operations, their computational complexity and memory footprint are substantial. Consequently, their inference speed is often far from meeting the real-time requirements of applications such as autonomous driving.

### 2.2. Voxel-Based Methods

Voxel-based methods leverage well-established Convolutional Neural Networks (CNNs) by converting the point cloud into a regular 3D voxel grid, thereby achieving a significant efficiency advantage when processing large-scale, unordered point clouds. VoxelNet [16] was a pioneering work in this direction, first proposing to partition the point cloud into voxels and perform per-voxel feature encoding. However, its reliance on dense 3D convolutions resulted in excessively high computational complexity. To address this issue, SECOND [17] innovatively introduced sparse 3D convolutions, which perform computations only on non-empty voxels, thus substantially improving processing speed. PointPillars [18] further simplified the point cloud into “pillars” along the vertical dimension and applied efficient 2D CNNs for feature extraction, achieving real-time inference performance. Building on these advancements, anchor-free detectors, exemplified by CenterPoint [19], have simplified the detection pipeline by directly predicting object centers. This approach has struck an excellent balance between accuracy and speed, establishing it as a widely adopted baseline model today.

However, the high efficiency of these methods comes at the cost of information fidelity. The feature encoding process within voxelization is, in essence, a form of lossy compression, aggregating all the geometric information of points within a local region into a single feature vector. For small objects, which are inherently represented by a sparse set of points, this loss of information is particularly severe and constitutes a major bottleneck for their detection performance.

### 2.3. Point-Voxel Fusion Methods

Point-voxel fusion methods endeavor to combine the advantages of the preceding two approaches. A quintessential example is PV-RCNN, which utilizes a voxel-based backbone network to efficiently generate high-quality 3D proposals [20]. Subsequently, a point-based branch network encodes the fine-grained geometric features of keypoints to refine the locations of these proposals. While this class of methods typically achieves high detection accuracy, their two-stage, complex architecture and the cross-representation feature interaction result in a substantial overall computational overhead and considerable deployment challenges.

In recent years, attention mechanisms and the Transformer architecture have also been introduced into the domain of point cloud processing [21]. For instance, Point Transformer [22] models local geometric relationships through self-attention mechanisms among points, whereas Voxel Transformer [23] captures the global context at the voxel level. Although these methods have demonstrated significant potential in feature modeling, they are often computationally expensive. Furthermore, when integrated with a voxelization front-end, they still face the persistent challenge of compensating for the initial loss of geometric information.

Most recently, deep learning paradigms for point cloud processing have witnessed significant advancements. For instance, Rehman et al. [24] provided a systematic review on the fusion of hyperspectral and LiDAR imagery, elucidating the critical transition from traditional machine learning to advanced CNNs. In a similar vein, Coglan et al. [25] demonstrated the efficacy of combining geometric feature analysis with deep learning, underscoring the importance of structural awareness. Furthermore, emerging works have focused on refining feature representations through attention mechanisms and voxel encoding. Specifically, Wang et al. [26] proposed a channel-wise attention network to dynamically prioritize informative features for 3D detection, while Naich et al. [27] introduced an intensity-aware voxel encoder to enhance robustness against environmental noise. Building upon these cutting-edge methodologies, our HFSA-Net integrates structured voxel encoding with a hybrid-domain attention mechanism to explicitly address the challenges of sparsity and scale variation.

In summary, all existing LiDAR-based 3D object detection methods negotiate a trade-off between computational efficiency and information completeness to varying degrees. Voxel-based methods, in their pursuit of real-time performance, sacrifice critical local geometric information during the preprocessing stage. Conversely, while point-based methods ensure information integrity, their prohibitive computational overhead restricts their application in real-world scenarios. The objective of this research is therefore to operate within the efficient paradigm of voxel-based frameworks, yet proactively enhance the critical information that is lost during data processing. We achieve this by developing purpose-built structured encoding and attention mechanisms, ultimately culminating in the construction of an enhanced 3D object detection network.

## 3. Methods

In this section, we present the detailed framework of the proposed HFSA-Net. We begin with an overview of the overall network architecture. Subsequently, we describe the three key components designed to handle point cloud sparsity: the structural feature encoder, the attention-guided backbone, and the multi-scale detection head. Finally, we introduce the loss function used for model training.

### 3.1. Overall Architecture

To address the challenges of existing anchor-free detectors in leveraging LiDAR point cloud structural information, focusing on key features, and perceiving multi-scale objects, we propose an enhanced 3D object detection framework: HFSA-Net (Hierarchical Focus and Structural-Aware Network). Built upon the CenterPoint architecture, this framework systematically improves the model’s overall performance by incorporating three specially designed, innovative components.

**Figure 1:**
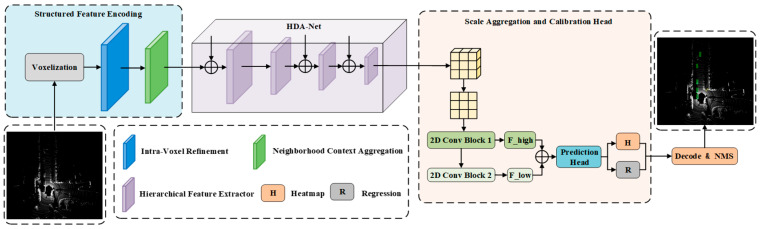
Block diagram of the proposed network.

As illustrated in Figure 1, the architecture of HFSA-Net is sequentially composed of a Structured Voxel Feature Encoder (S-VFE), a Hybrid-Domain Attention-guided sparse Backbone (HDA-Backbone), and a Scale-Aggregation Head (SA-Head), which replace their corresponding modules in the original CenterPoint. The entire data processing pipeline begins with the S-VFE performing structured encoding on the raw point cloud, followed by the HDA-Backbone conducting hierarchical focused extraction on the structured features. Finally, the SA-Head performs multi-scale decoding and prediction in the 2D Bird’s-Eye View (BEV) space. The design of each component will be elaborated below.

### 3.2. Structured Voxel Feature Encoder

When processing LiDAR point clouds, traditional Voxel Feature Encoder (VFE) methods simplify voxelization to the mean of features of the points within each voxel, leading to an irreversible loss of information. This loss is twofold: first, micro-geometric structures, such as the intra-voxel point distribution, are obliterated; second, macro-contextual information, which is jointly constituted by adjacent voxels, is completely disregarded. To this end, we design the Structured Voxel Feature Encoder (S-VFE), which aims to re-encode this rich structural information back into the voxel features through a two-stage process.

**Figure 2:**
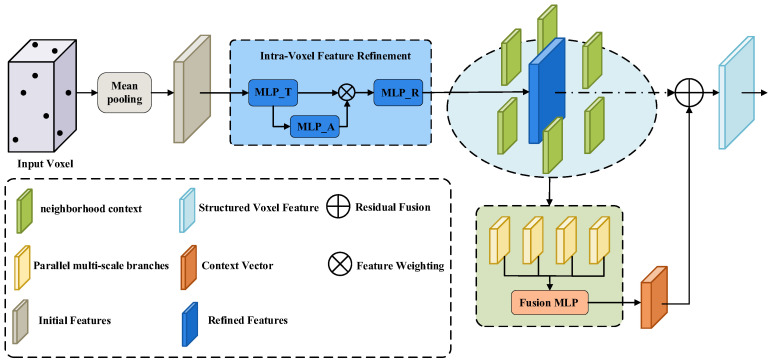
Block diagram of the structured voxel feature encoder.

As depicted in Figure 2, the first stage of the S-VFE is intra-voxel feature refinement. For a given voxel v with an initial mean feature $f_{v}$, we first compute a refined feature $f_{v}^{ref}$ by passing it through a feature transformation network $T_{\mathrm{MLP}}$ and an attention network ${\mathrm{Attn}}_{\mathrm{MLP}}$. This process utilizes the Hadamard Product (⊙) to perform feature weighting:

(1) $$w_{v}=\sigma ({\mathrm{Attn}}_{\mathrm{MLP}}(T_{\mathrm{MLP}}(f_{v})))$$

(2) $$f_{v}^{\mathrm{ref}}=T_{\mathrm{MLP}}(f_{v})⊙w_{v}$$

In the expressions above, $T_{\mathrm{MLP}}$ and ${\mathrm{Attn}}_{\mathrm{MLP}}$ are both small Multi-Layer Perceptrons (MLPs), and σ denotes the Sigmoid activation function.

The second stage is multi-scale neighborhood context aggregation. In this work, we treat all non-empty voxels as nodes of an implicit graph. For each voxel $v_{i}$, information from its $k_{m}$ nearest neighbors, $N_{k_{m}}(v_{i})$, is aggregated in parallel across M different scales. The context feature $c_{i}^{m}$ at each scale is computed by a scale-specific MLP processor, $P_{m}$:

(3) $$c_{i}^{m}=P_{m}\left(\frac{1}{k_{m}}\sum_{v_{j}\in N_{k_{m}}(v_{i})}f_{v_{j}}^{\mathrm{ref}}\right)$$

In the specific implementation of HFSA-Net, the voxel resolution is configured as [0.05 m, 0.05 m, 0.1 m] to ensure sufficient granularity for small objects such as pedestrians. For the neighborhood aggregation, we employ M = 4 parallel scales with neighbor counts set to k ∈ {4, 8, 16, 32}. This multi-scale design allows the encoder to simultaneously capture fine local geometry and broad contextual information to compensate for sparsity.

After the context features from all scales, ${\left\{c_{i}^{m}\right\}}_{m=1}^{M}$, are concatenated, they are fed into a fusion network, $F_{\mathrm{fuse}}$. The final output feature, $f_{v_{i}}^{\mathrm{out}}$, is formed via a residual connection [28], where λ is a learnable scaling parameter:

(4) $$f_{v_{i}}^{\mathrm{out}}=f_{v_{i}}^{\mathrm{ref}}+\lambda \cdot F_{\mathrm{fuse}}\left(\underset{m=1..M}{Concat}[c_{i}^{m}]\right)$$

Through the S-VFE, the output feature of each voxel not only contains its own refined information but also explicitly encodes its position and relationships within the multi-scale local geometric structure.

### 3.3. Hybrid-Domain Attention-Guided Sparse Backbone

The inherent sparsity of a LiDAR point cloud leads to the formation of a vast number of empty voxels after voxelization. Although sparse convolutional networks can enhance efficiency by skipping computations on these empty voxels, they, by default, assign equal importance to all activated voxels. However, in real-world LiDAR perception scenarios, critical foreground objects such as distant pedestrians or small obstacles may be represented by only a few isolated, activated voxels. Standard networks struggle to effectively focus on these faint yet crucial foreground signals amidst a multitude of background voxels. To address this, we propose the Hybrid-Domain Attention-guided sparse Backbone (HDA-Backbone). The core idea is to decouple the attention mechanism into two orthogonal feature domains: the spatial domain, to locate critical regions, and the channel domain, to select key semantics.

**Figure 3:**
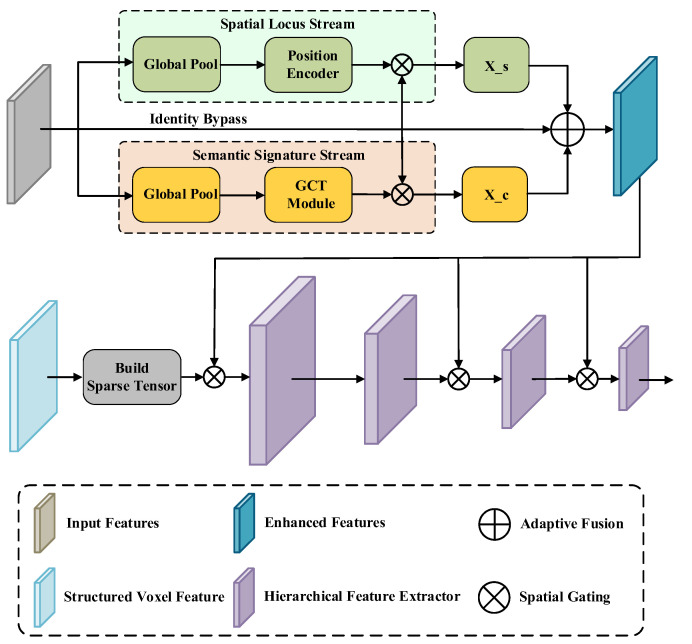
Block diagram of the hybrid-domain attention-guided sparse backbone network.

As illustrated in Figure 3, the core of the HDA-Backbone is the Decoupled Hybrid Attention (DHA) module. For an input sparse feature tensor X, this module concurrently computes a spatial attention map, $M_{s}$, generated by the Fast Coordinate Attention (FastCA) [29] mechanism, and a channel attention map, $M_{c}$, produced by the lightweight Gated Channel Transformation (GCT) [30] module. Here, FastCA is selected over standard global pooling methods, such as the SE-Block, to explicitly preserve the spatial structure along coordinate axes. Meanwhile, GCT is chosen for its efficiency, as it filters semantic features using a normalization-based gating mechanism without the heavy computational burden of complex self-attention layers. These two attention maps are adaptively fused via a learnable gating parameter, α, and are applied to the original features in a residual manner to obtain the enhanced features $X^{\prime }$:

(5) $$X^{\prime }=X+\alpha \cdot (X⊙M_{s})+(1-\alpha )\cdot (X⊙M_{c})$$

where $M_{s}$ and $M_{c}$ are computed by FastCA and GCT, respectively, and the symbol ⊙ denotes element-wise multiplication (Hadamard product). The parameter α, implemented via a Sigmoid activation function, serves as an adaptive gating coefficient. Its primary role is to dynamically govern the trade-off between geometric and semantic information. During training, the network automatically adjusts α via back-propagation; specifically, a higher value prioritizes spatial structural cues derived from FastCA, whereas a lower value emphasizes semantic channel features extracted by GCT, thereby optimizing the feature representation layer-by-layer.

Furthermore, considering that features extracted at different network depths possess varying semantic levels, we adopt a hierarchical integration strategy. The DHA module is strategically embedded at the entrance of the backbone network and after each downsampling stage. This ensures that features throughout the entire pipeline receive dynamic and appropriate focus and enhancement.

### 3.4. Scale-Aggregation Head

The final detection head confronts a core real-world challenge: the significant variation in object scales within autonomous driving scenes. A standard single-scale detection head, constrained by its fixed receptive field, struggles to efficiently and simultaneously handle both large, nearby vehicles and small, distant pedestrians.

**Figure 4:**
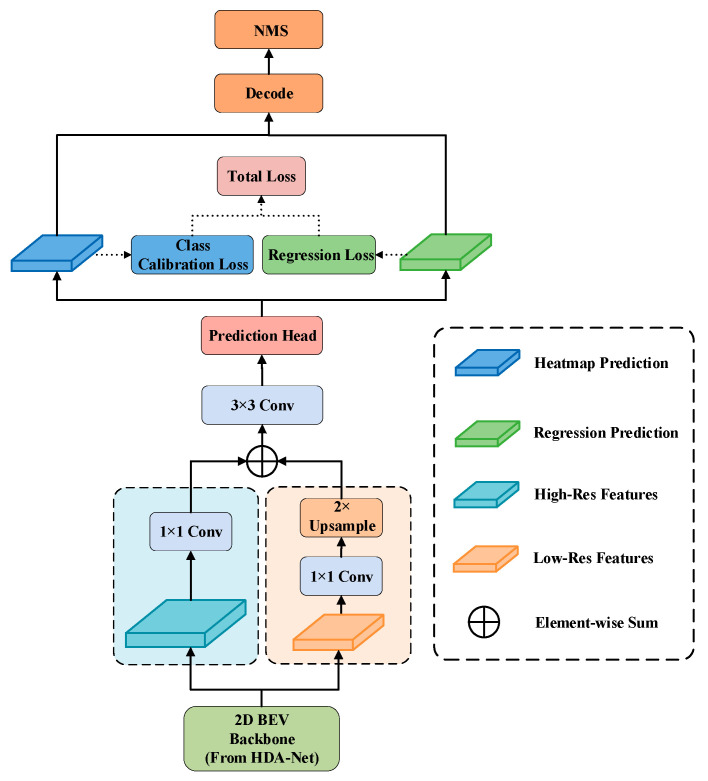
Block diagram of the scale-aggregated detection head.

To address the issue of non-uniform density distribution in LiDAR point clouds, we designed the Scale-Aggregation Head (SA-Head), the core of which lies in explicitly handling multi-scale information through its structural design. As shown in Figure 4, the SA-Head introduces a multi-scale feature aggregation mechanism [31]. It receives high-resolution features, $F_{h}$, and low-resolution features, $F_{l}$, from the 2D BEV backbone. A fusion module, akin to a Feature Pyramid Network (FPN) [32,33], integrates these two feature types to generate a scale-complete, unified feature plane, $F_{\mathrm{agg}}$. This is achieved through lateral connections, $\phi_{h}$ and $\phi_{l}$, implemented by 1 × 1 convolutional layers, and an upsampling operator, U:

(6) $$F_{\mathrm{agg}}=\phi_{h}(F_{h})+\phi_{l}(U(F_{l}))$$

$F_{\mathrm{agg}}$ is subsequently fed into shared convolutional layers and separate prediction heads for heatmap prediction and bounding box attribute regression, respectively. In this manner, the SA-Head can leverage information from different semantic levels within a single decoding stage. This provides a solid structural foundation for concurrently detecting objects of various sizes, thereby significantly enhancing the model’s overall adaptability to complex scenes.

### 3.5. Loss Function

We employ a multi-task loss function, L, to train the network. This loss function is composed of a classification loss for supervising the center point heatmap prediction and a regression loss for optimizing the 3D bounding box attributes.

For the classification task, we adopt the Focal Loss [34], denoted as $L_{fl}$, which is widely used in dense object detection. In point cloud detection scenarios, there is a severe foreground-background imbalance, as the number of object centers is far less than the number of background locations. Focal Loss effectively addresses this issue by introducing a modulating factor that down-weights the contribution of a large number of easy negative samples to the total loss, thereby enabling the model to focus more on learning from hard positive samples. For the regression of the various 3D bounding box attributes, we utilize the Smooth L1 Loss [35], denoted as $L_{reg}$. This loss is applied only at the locations corresponding to the ground-truth object centers (derived from the high-precision KITTI benchmark annotations [2]) and computes the deviation between the predicted values and the true values.

The total loss of the network, L, is a weighted sum of the aforementioned classification and regression losses. We introduce a balancing hyperparameter, $\lambda_{reg}$, to adjust the weight of the regression loss. The total loss is calculated as follows:

(7) $$L=L_{fl}+\lambda_{reg}L_{reg}$$

## 4. Experiments and Result Analysis

To comprehensively validate the effectiveness and robustness of HFSA-Net, extensive experiments were conducted on the KITTI benchmark. This section first details the dataset characteristics, implementation parameters, and evaluation metrics. Subsequently, we present a quantitative comparison with mainstream 3D object detection methods, followed by an ablation study that dissects the specific contributions of each proposed module.

### 4.1. Dataset

All experiments in this study were conducted on the widely used KITTI dataset. The data acquisition platform for this dataset is equipped with grayscale cameras, color cameras, and a Velodyne HDL-64E LiDAR sensor. This Velodyne LiDAR features 64 scan lines and captures high-precision 3D point cloud data at a frequency of 10 Hz. Key technical specifications of this sensor are summarized in Table 1. The KITTI dataset comprises a total of 7481 samples, covering three core categories: ‘Car’, ‘Pedestrian’, and ‘Cyclist’. Following the widely adopted standard data split protocol proposed by Chen et al. [36], we split the official training set (7481 samples) into a training set of 3712 samples and a validation set of 3769 samples. This division helps to avoid overfitting and ensures a fair comparison with other methods using the same configuration. These sets were used for model training and ablation studies, respectively.

### 4.2. Experimental Setup and Parameters

Implementation Details. Our research was conducted based on the OpenPCDet framework, an open-source toolbox for 3D object detection. The experimental environment was configured as follows: the operating system was Ubuntu 20.04, a Python 3.8 virtual environment was created using Anaconda3, and the training framework was built upon PyTorch 1.10.0 with CUDA 11.1. The hardware platform consisted of an Intel Core i5-12490F CPU (Intel Corporation, Santa Clara, CA, USA) and an NVIDIA GeForce RTX3060 GPU (NVIDIA Corporation, Santa Clara, CA, USA).

Training Parameters. During the training process, the batch size was set to 4 due to the memory constraints of the single NVIDIA GeForce RTX 3060 GPU. The model was trained for 100 epochs. We used the Adam optimizer combined with the OneCycle scheduling policy to ensure stable convergence. The initial learning rate was set to 0.002, which was adjusted based on the Linear Scaling Rule to align with the reduced batch size. The weight decay was set to 0.01, and gradient clipping (with a norm of 10) was applied to prevent exploding gradients. For the enhanced attention modules, their learning rate was set to 1/10 of that of the backbone network to ensure stability in feature extraction. Data Processing. For data processing, the point cloud range was defined as [0, −40, −3] to [70.4, 40, 1] along the x, y, and z axes, respectively. The voxel size was set to (0.05, 0.05, 0.1) meters. The maximum number of voxels was configured to 16,000 during training and 40,000 during inference. To improve the model’s generalization ability, we employed several data augmentation strategies during training, including: (1) random horizontal flipping with a probability of 0.5; (2) random rotation around the vertical axis within a range of [−45°, +45°]; and (3) global scaling with a random factor between 0.95 and 1.05 to enrich sample diversity.

### 4.3. Evaluation Metrics

We adopt the official evaluation metrics of the KITTI benchmark, namely Average Precision (AP), which is calculated using 40 recall positions. The evaluation is conducted from three perspectives: 3D detection, Bird’s-Eye View (BEV) detection, and Average Orientation Similarity (AOS).

According to the official KITTI criteria, the Intersection over Union (IoU) threshold is set to 0.7 for the ‘Car’ category and 0.5 for the ‘Pedestrian’ and ‘Cyclist’ categories. For each category, the AP is reported for three difficulty levels: Easy, Moderate, and Hard. The AP is defined as:

(8) $$AP_{R40}=\frac{1}{\left|R_{40}\right|}\sum_{r\in R_{40}}p_{\mathrm{interp}}(r)$$

(9) $$p_{\mathrm{interp}}(r)=\max_{\tilde{r}:\tilde{r}\geq r}p(\tilde{r})$$

$R_{40}$ is a set containing 40 equally spaced recall thresholds, $\left|R_{40}\right|$ is the cardinality of this set, and $p_{\mathrm{interp}}(r)$ is the interpolated precision at a recall level r. $p(\tilde{r})$ denotes the measured precision at an actual recall of ř. The AOS is also sampled at these 40 recall positions, and its formula is defined as:

(10) $$AOS_{R40}=\frac{1}{\left|R_{40}\right|}\sum_{r\in R_{40}}s_{\mathrm{interp}}(r)$$

(11) $$s_{\mathrm{interp}}(r)=\max_{\tilde{r}:\tilde{r}\geq r}s(\tilde{r})$$

(12) $$s(\tilde{r})=\frac{1}{\left|D(\tilde{r})\right|}\sum_{i\in D(\tilde{r})}\frac{1+\cos(\Delta \theta^{\left(i\right)})}{2}\cdot \delta_{i}$$

where $s_{\mathrm{interp}}(r)$ is the interpolated orientation similarity at recall level r, and $s(\tilde{r})$ is the average orientation similarity of all True Positive (TP) detections at a recall of $\tilde{r}$. $D(\tilde{r})$ is the set of all detections classified as TP at recall $\tilde{r}$, and $\left|D(\tilde{r})\right|$ is the number of elements in this set. $\Delta \theta^{\left(i\right)}$ represents the angle error between the estimated orientation and the ground-truth orientation for the i-th detection.

### 4.4. Experimental Result Analysis

#### 4.4.1. Loss Curve

**Figure 5:**
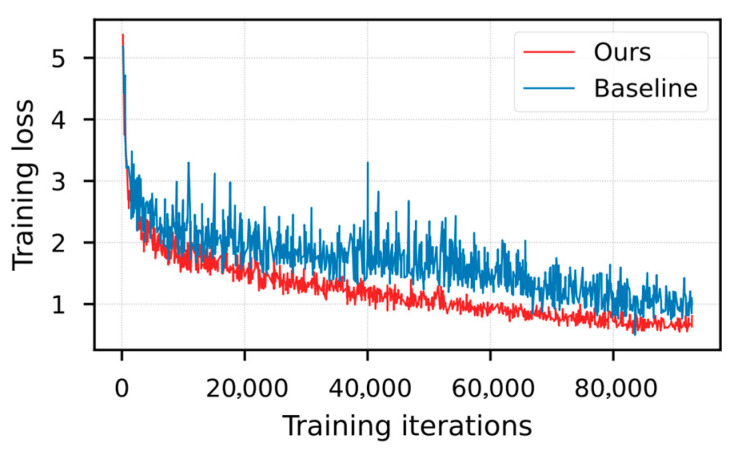
Curve of total loss versus time.

During the training process of this experiment, the trend of the total loss value over time is illustrated in Figure 5. Specifically, on the experimental platform (RTX 3060 GPU), the average time per iteration (batch size = 4) is approximately 0.48 s, and the total training time for 100 epochs is about 12.5 h. It is evident that the proposed algorithm maintains a lower loss value throughout the entire training process.

#### 4.4.2. Quantitative Analysis

To evaluate the effectiveness of our proposed model, we conducted a comprehensive comparison with mainstream 3D object detection methods. Specifically, CenterPoint [19] was selected as the primary baseline to verify the architectural improvements. We also included foundational voxel-based methods (VoxelNet [16], SECOND [17]) and the widely used real-time detector PointPillars [18] to benchmark against classic standards. Additionally, F-PointNet represents the point-based paradigm, providing a cross-category comparison. The results are presented in Table 2, Table 3 and Table 4.

3D detection performance is a core metric for assessing a model’s comprehensive perception capability. As shown in Table 3, HFSA-Net achieves an mAP of 66.93%, outperforming all comparative methods and marking an improvement of 3.34% over the CenterPoint baseline. Most notably, on the highly challenging pedestrian detection task, our model attains an AP of 51.17%, representing a substantial 6.04% gain over the baseline. This significant uplift demonstrates the effectiveness of the proposed S-VFE module in encoding fine-grained geometric structures, as well as the advantage of the SA-Head in fusing high-resolution features. Furthermore, the robust improvements in the ‘Car’ and ‘Cyclist’ categories indicate the general applicability of our proposed algorithmic framework.

The BEV detection performance reflects the model’s capabilities in 2D planar localization and size estimation. As presented in Table 2, HFSA-Net also exhibits outstanding performance from this perspective, achieving an mAP of 72.64% and comprehensively surpassing methods like CenterPoint and PointPillars. This indicates that our proposed modules, particularly the attention-enhanced features provided by the HDA-Backbone and the multi-scale fusion from the SA-Head, have effectively improved the quality of feature representation in the bird’s-eye view.

The AOS performance provides a composite evaluation of 2D detection and 3D orientation estimation. Our model achieves an mAP of 80.76% in this metric, which is also superior to the CenterPoint baseline. This demonstrates that the rich structural and semantic features extracted by our network are also beneficial for accurately predicting the 3D orientation of objects.

#### 4.4.3. Qualitative Analysis

**Figure 6:**
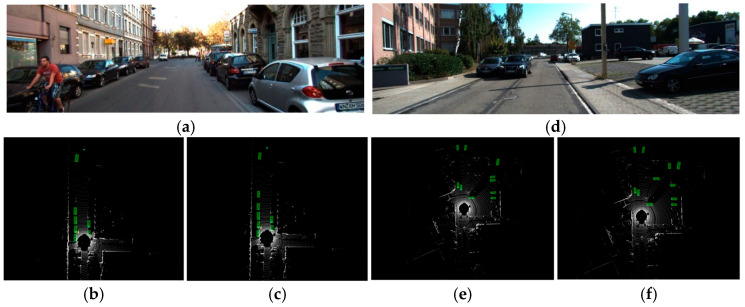
Detection results of the two algorithms in different scenes. (**a**) Original photo of Scene 1; (**b**) Detection result of the baseline method for Scene 1; (**c**) Detection result of the proposed method for Scene 1; (**d**) Original photo of Scene 2; (**e**) Detection result of the baseline method for Scene 2; (**f**) Detection result of the proposed method for Scene 2.

To provide a more intuitive demonstration of HFSA-Net’s performance, we present a qualitative comparison of detection results on the KITTI dataset in Figure 6. The figure displays two challenging scenarios that include distant small objects and partially occluded vehicles. It can be clearly observed that in regions where the baseline model, CenterPoint, either misses objects or provides inaccurate localization, HFSA-Net is able to detect the targets successfully and precisely. For instance, in the first scenario, HFSA-Net successfully identifies a pedestrian in an occluded area, whereas the baseline model completely overlooks this target. These visual results provide strong corroborating evidence for our quantitative analysis.

### 4.5. Ablation Study

To thoroughly investigate the effectiveness of the three proposed modules and their interactions, we designed a series of detailed ablation studies. Starting from the CenterPoint baseline model (denoted as the first row in the tables), we progressively integrated our innovative components. In the tables, S-VFE represents the Structured Voxel Feature Encoder, HDA stands for the Hybrid-Domain Attention-guided sparse Backbone, and SA-H signifies the Scale-Aggregation Head. The experimental results were evaluated from the BEV, 3D, and AOS perspectives, as presented in Table 5, Table 6 and Table 7.

First, we evaluated the individual contribution of each module. When SA-H was introduced alone, the model achieved the largest individual gain of 2.66% in 3D mAP, primarily attributed to its significant improvements on the ‘Car’ and ‘Cyclist’ categories. This advantage was equally evident from the BEV and AOS perspectives, which strongly demonstrates that multi-scale fusion is crucial for enhancing object localization and size estimation. The individual addition of S-VFE led to a 0.51% increase in 3D mAP, with its main advantage being the refined perception of the ‘Pedestrian’ category. Although its overall gains in BEV and AOS were modest, it provided higher-quality initial features for subsequent modules. Introducing HDA alone yielded a 0.21% improvement in 3D mAP, indicating that its effect is limited without the cooperation of other modules. However, it laid the foundation for subsequent synergistic enhancements.

Second, we assessed the synergistic effects between the modules. The performance of module combinations surpassed the sum of their individual contributions. Among them, the combination of HDA and SA-H was particularly outstanding, achieving a 3.76% improvement in 3D mAP, along with gains of 1.06% in BEV mAP and 0.42% in AOS mAP. This reveals that a powerful attention mechanism (HDA) provides high-quality features for an advanced multi-scale head (SA-H), thereby maximizing its fusion capabilities. This demonstrates the complementary nature of the proposed modules: the S-VFE enriches the input geometric representation, the HDA-Backbone refines feature selection, and the SA-Head ensures robust multi-scale perception. Together, they optimize the entire detection pipeline from encoding to prediction.

Finally, we evaluated the complete model, HFSA-Net (S-VFE + HDA + SA-H). With the support of all modules, it delivered the most comprehensively powerful and robust solution. From the 3D perspective, it achieved a 3.34% mAP improvement over the baseline. From the BEV perspective, the mAP gain was 2.77%, and from the AOS perspective, it was 1.23%. The consistent and significant performance growth across these three core evaluation dimensions uniformly validates the comprehensiveness and effectiveness of our proposed enhanced framework. The final model also demonstrated stronger robustness on other metrics, such as the ‘Hard’ difficulty level, confirming the unique value of S-VFE in processing sparse and challenging samples.

### 4.6. Real-Vehicle Experiment

**Figure 7:**
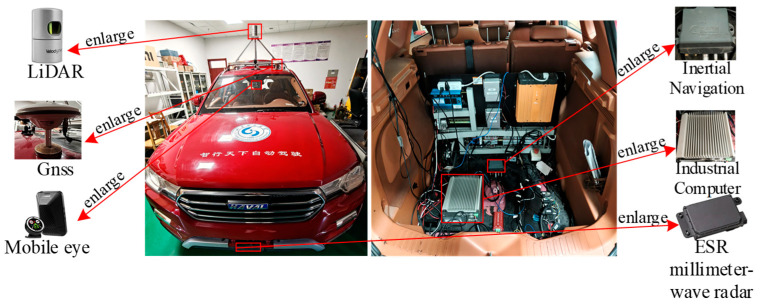
Vehicles used in the experiment.

To validate the adaptability and robustness of our proposed object detection algorithm in a real-world traffic environment, we conducted a series of real-vehicle experiments. Our experimental platform, as shown in Figure 7, is an autonomous research vehicle modified from a Haval H7. The vehicle is equipped with a Velodyne HDL-32E LiDAR sensor. The key technical specifications of this sensor are detailed in Table 8.

Compared to the Velodyne HDL-64E used to create the KITTI dataset, the HDL-32E has fewer vertical scan lines and a lower point cloud output rate, meaning the point clouds it generates are inherently sparser. Additionally, the vehicle is outfitted with other core equipment, including cameras, a combined GPS-IMU navigation system, and an industrial control computer. The installation positions and layout of the sensors are also depicted in Figure 7.

**Figure 8:**
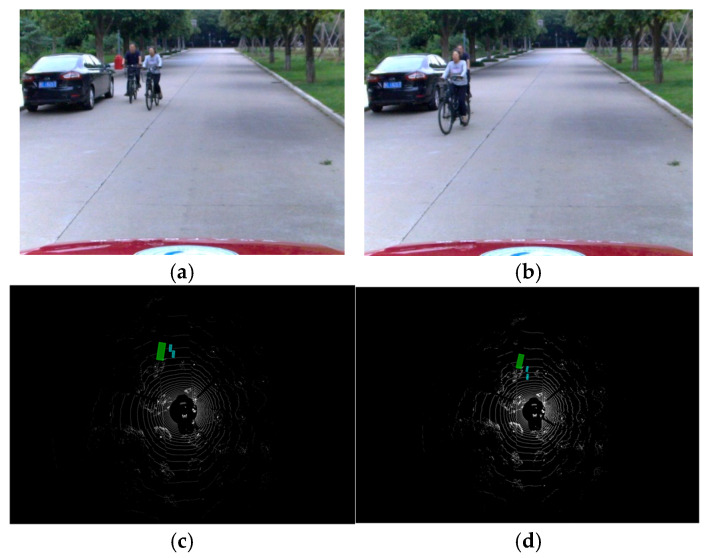
Detection results of the real-vehicle experiment. (**a**) Original photo of Scene 1; (**b**) Original photo of Scene 2; (**c**) Detection result of the proposed method for Scene 1; (**d**) Detection result of the proposed method for Scene 2.

We collected traffic scenes from a campus road environment with the vehicle moving at a safe speed of approximately 20–30 km/h, and processed the raw data packets into a standardized dataset compatible with the KITTI format. Although vehicle motion theoretically introduces point cloud distortion, at this low speed, such distortion is negligible and has a minimal impact on detection accuracy. Therefore, the primary challenge stems from the relatively low resolution of the vehicle-mounted LiDAR and environmental factors such as occlusions, which result in a point cloud density significantly lower than that of the KITTI dataset. As shown in Figure 8, despite these challenges, the improved algorithm demonstrates strong detection capabilities.

As depicted in Figure 8c displays the detection results in a campus scene, where the model is able to accurately identify multi-class traffic participants. In panel (d), despite occlusions between a cyclist and a vehicle and the resulting point cloud sparsity, our algorithm still successfully distinguishes between the different targets. Furthermore, the experiment validates the model’s robustness against dynamic targets. Despite the cyclist moving at speeds of approximately 10–15 km/h, the proposed S-VFE module effectively captures the structural integrity of the point cloud, which remains largely unaffected by motion distortion at these velocities. Moreover, the model accurately predicts the heading angle across varying orientations. This capability is quantitatively corroborated by the high Average Orientation Similarity (AOS) of 80.76% reported in Table 4, confirming the network’s effectiveness in perceiving moving objects with diverse velocities and trajectories.

The experiment demonstrates that the proposed method is not reliant on a specific high-density sensor configuration. It possesses excellent cross-sensor generalization ability and can effectively address the detection challenges in complex traffic scenarios.

## 5. Discussion

In this section, we provide a critical analysis of the experimental findings. We first interpret the underlying reasons for the performance improvements, particularly regarding small and sparse objects. We then examine the model’s generalization capabilities across different sensor configurations. Finally, we discuss the computational trade-offs and current limitations to suggest directions for future research.

### 5.1. Performance Analysis and Interpretations

The experimental results on the KITTI dataset demonstrate that HFSA-Net effectively addresses the challenges of point cloud sparsity. With a 3D mAP of 66.93%, our model outperforms the baseline CenterPoint by 3.34%. A critical finding is the significant improvement in the ‘Pedestrian’ category (+6.04% AP). Pedestrians occupy very few voxels in LiDAR scans, making them highly sensitive to the quantization artifacts caused by standard voxelization. The success of HFSA-Net in this category is attributed to the synergy between the high voxel resolution and the Structured Voxel Feature Encoder (S-VFE). The fine resolution ensures sufficient spatial extent for small objects, while the S-VFE employs neighbor aggregation to preserve fine-grained intra-voxel details that are typically discarded by mean-pooling operations.

### 5.2. Generalization and Practicality

The real-vehicle experiments provide crucial insight into the model’s robustness. While the model was trained on the high-resolution KITTI dataset (64-beam), it was tested on a vehicle platform equipped with a lower-resolution Velodyne HDL-32E sensor (32-beam). Despite the domain gap and reduced point density, HFSA-Net successfully detected cyclists and pedestrians without fine-tuning. This indicates that the Scale-Aggregation Head (SA-Head) effectively mitigates the impact of varying point densities by fusing multi-scale features, making the algorithm highly adaptable for deployment on autonomous vehicles with varying sensor configurations.

### 5.3. Limitations and Future Work

Despite the promising results, this study presents certain limitations. First, regarding computational cost, the proposed S-VFE module utilizes multi-scale neighborhood aggregation, which introduces additional latency compared to simple mean-pooling operations. However, experimental evaluation on an RTX 3060 GPU demonstrates an average inference latency of 42.8 ms, corresponding to a speed of approximately 23.4 FPS. This processing speed meets the real-time constraints of standard autonomous driving systems that typically operate at 10 Hz. Second, the current experiments were conducted primarily in clear weather scenarios. Future work will focus on extending the framework to multi-modal fusion by integrating LiDAR and camera data. Specifically, we aim to incorporate image-derived semantic information into the S-VFE module to enrich point representation. Additionally, the attention mechanisms within the HDA-Backbone will be adapted to fuse cross-modal features, thereby enhancing detection robustness in adverse weather conditions where point cloud noise increases significantly.

## 6. Conclusions

In response to the limitations imposed on 3D object detection by the inherent sparsity and geometric structural fragility of LiDAR point clouds, this paper has proposed an enhanced 3D object detection framework: HFSA-Net. First, our designed Structured Voxel Feature Encoder (S-VFE) effectively overcomes the information loss associated with traditional VFE methods through intra-voxel refinement and multi-scale neighborhood context aggregation. Second, the constructed Hybrid-Domain Attention-guided sparse Backbone (HDA-Backbone) utilizes a decoupled hybrid attention mechanism optimized for sparse data, enabling the network to adaptively focus on critical features and thereby enhancing both the efficiency and quality of feature extraction. Finally, the proposed Scale-Aggregation Head (SA-Head) significantly improves the model’s perception capabilities for objects of varying sizes by fusing multi-scale BEV features. Extensive experiments conducted on the public KITTI dataset have thoroughly validated the effectiveness of our proposed method. Compared to the CenterPoint baseline, HFSA-Net improves the 3D mean Average Precision (mAP) by 3.34%. It achieves a remarkable increase of up to 6.04% in average precision on the challenging ‘Moderate’ difficulty pedestrian detection task, which demonstrates HFSA-Net’s superior performance in handling sparse, small-sized targets. The general applicability and robustness of the framework were further confirmed through ablation studies and real-vehicle experiments. Future work will explore extending this framework to multi-modal data fusion scenarios to further enhance 3D perception capabilities in complex scenes.

## Figures and Tables

**Table 1:** Key technical specifications of the LiDAR sensor used in the KITTI dataset.

Parameters	Values
Laser Harness (Wire)	64
Measuring range (m)	120
Range Accuracy (cm)	2
Horizontal FoV (°) Vertical FoV (°)	360 26.8
Output (points per second)	1,300,000

**Table 2:** The detection accuracy of different algorithms on the dataset from the perspective of BEV (%).

Model	Car			Pedestrian			Cyclist			mAP
	Easy	Mode	Hard	Easy	Mode	Hard	Easy	Mode	Hard	
Second	88.07	84.00	75.33	58.09	50.22	47.20	83.66	66.19	62.13	68.24
VoxelNet	89.35	79.26	77.39	46.13	40.74	38.11	66.7	54.76	50.55	60.33
F-PointNet	88.70	84.00	75.33	58.09	51.05	47.54	75.38	61.69	54.68	66.27
PointPillars	92.05	87.80	85.19	56.53	50.83	46.43	81.32	65.07	60.73	69.55
Centerpoint	91.41	85.63	83.04	56.02	51.77	47.96	80.10	68.11	64.80	69.87
Ours	91.31	87.62	86.06	62.16	57.06	52.58	83.19	68.93	64.83	72.64

**Table 3:** The detection accuracy of different algorithms in the 3D perspective of the dataset (%).

Model	Car			Pedestrian			Cyclist			mAP
	Easy	Mode	Hard	Easy	Mode	Hard	Easy	Mode	Hard	
Second	86.44	76.97	73.39	47.47	40.47	36.26	81.28	63.49	59.29	62.78
VoxelNet	77.47	65.11	57.73	39.48	33.69	31.50	61.22	48.36	44.37	50.99
F-PointNet	81.20	70.39	62.19	51.21	44.89	40.23	71.96	56.77	50.39	58.80
PointPillars	85.03	75.76	72.74	50.08	44.18	39.53	77.13	60.94	56.91	62.48
Centerpoint	86.86	75.98	73.09	49.70	45.13	41.16	76.73	63.34	60.34	63.59
Ours	85.80	77.63	75.46	56.18	51.17	46.85	82.39	65.01	61.90	66.93

**Table 4:** The detection accuracy of different algorithms from the perspective of AOS on the dataset (%).

Model	Car			Pedestrian			Cyclist			mAP
	Easy	Mode	Hard	Easy	Mode	Hard	Easy	Mode	Hard	
Second	94.84	90.94	90.11	60.01	53.92	50.77	89.40	72.82	68.87	74.63
SubCNN	90.61	88.43	78.63	78.33	66.28	61.37	71.39	63.41	46.34	71.64
AVOD-FPN	89.95	87.13	79.74	53.36	44.92	43.77	67.61	57.53	54.16	64.24
PointPillars	95.02	91.24	88.46	47.33	44.40	41.31	84.75	71.35	67.24	70.12
Centerpoint	95.56	89.73	88.95	70.10	65.31	61.61	90.59	78.68	75.25	79.53
Ours	96.03	92.52	90.36	74.35	69.59	65.21	90.01	76.38	72.34	80.76

**Table 5:** The detection accuracy of the ablation experiment from the BEV perspective on the KITTI dataset (%).

S-VFE	HDA	SA-H	Car			Pedestrian			Cyclist			mAP
			Easy	Mode	Hard	Easy	Mode	Hard	Easy	Mode	Hard	
			91.41	85.63	83.04	56.02	51.77	47.96	80.10	68.11	64.80	69.87
√			89.79	86.16	85.58	55.62	51.88	47.83	81.13	68.71	64.93	70.18
	√		89.34	86.08	85.61	56.65	51.78	47.72	83.53	67.80	63.60	70.23
		√	89.89	86.40	86.01	58.98	53.27	48.90	84.49	70.64	66.17	71.64
√	√		90.07	86.66	85.98	58.87	53.81	49.30	82.04	70.25	66.07	71.45
√		√	91.99	87.94	86.33	59.53	54.07	50.02	82.46	67.80	64.16	71.59
	√	√	89.61	87.64	86.16	60.93	56.54	51.51	82.07	70.71	66.73	72.44
√	√	√	91.31	87.62	86.06	62.16	57.06	52.58	83.19	68.93	64.83	72.64

**Table 6:** The detection accuracy of the ablation experiment in the 3D perspective on the KITTI dataset (%).

S-VFE	HDA	SA-H	Car			Pedestrian			Cyclist			mAP
			Easy	Mode	Hard	Easy	Mode	Hard	Easy	Mode	Hard	
			86.86	75.98	73.09	49.70	45.13	41.16	76.73	63.34	60.34	63.59
√			84.09	76.02	74.02	51.20	46.92	42.61	78.19	63.02	59.15	63.91
	√		85.49	75.91	74.07	50.83	45.97	41.55	81.54	63.18	59.04	64.18
		√	84.41	77.83	75.85	53.44	47.68	43.35	81.70	66.92	62.59	65.97
√	√		86.49	76.46	74.47	54.01	49.31	44.30	79.17	66.69	62.69	65.96
√		√	85.63	78.15	74.70	53.19	48.09	44.22	79.54	62.98	59.32	65.09
	√	√	84.95	77.29	75.63	57.17	52.40	47.45	77.90	66.03	61.95	66.75
√	√	√	85.80	77.63	75.46	56.18	51.17	46.85	82.39	65.01	61.90	66.93

**Table 7:** The detection accuracy of the ablation experiment from the AOS perspective on the KITTI dataset (%).

S-VFE	HDA	SA-H	Car			Pedestrian			Cyclist			mAP
			Easy	Mode	Hard	Easy	Mode	Hard	Easy	Mode	Hard	
			95.56	89.73	88.95	70.10	65.31	61.61	90.59	78.68	75.25	79.53
√			94.80	90.84	90.06	68.61	65.45	62.35	88.63	77.82	73.39	79.11
	√		94.30	92.06	90.23	71.68	67.10	62.63	89.22	75.72	72.23	79.47
		√	94.71	90.78	90.10	70.85	66.54	62.09	91.20	77.45	72.81	79.61
√	√		94.82	91.02	90.34	72.36	67.86	62.94	90.90	78.09	74.19	80.28
√		√	94.75	92.02	90.20	71.50	67.37	63.57	88.85	76.82	73.52	79.84
	√	√	94.48	92.46	90.24	71.26	67.84	64.40	88.51	77.31	73.11	79.95
√	√	√	96.03	92.52	90.36	74.35	69.59	65.21	90.01	76.38	72.34	80.76

**Table 8:** Key Technical Specifications of Velodyne HDL-32E.

Parameters	Values
Laser Harness (Wire)	32
Measuring range (m)	80–100
Range Accuracy (cm)	±2
Dimensions (mm)	85 × 144
Horizontal FoV (°)	360
Vertical FoV (°)	+10.67° to −30.67°
Supply Voltage (VDC)	9–32
Laser Class	Class 1
Power (W)	31.4
Output (points per second)	700,000

## Data Availability

The authors declare that upon reasonable request, the data and the code are available from the corresponding author.

## Abbreviations

The following abbreviations are used in this manuscript:

HFSA-Net	Hierarchical Focus and Structural-Aware Network
S-VFE	Structured Voxel Feature Encoder
HDA-Backbone	Hybrid-Domain Attention-guided sparse Backbone
SA-Head	Scale-Aggregation Head
FastCA	Fast Coordinate Attention
GCT	Gated Channel Transformation
FPN	Feature Pyramid Network
AP	Average Precision
AOS	Average Orientation Similarity
TP	True Positive
